# Predation risk in relation to brain size in alternative prey of pygmy owls varies depending on the abundance of main prey

**DOI:** 10.1371/journal.pone.0236155

**Published:** 2020-09-11

**Authors:** Anders Pape Møller, Kari Hongisto, Erkki Korpimäki

**Affiliations:** 1 Ecologie Systématique Evolution, Université Paris-Sud, CNRS, AgroParisTech, Université Paris-Saclay, France; 2 Tieteenkatu, Tampere, Finland; 3 Section of Ecology, Department of Biology, University of Turku, Turku, Finland; University of Western Australia, AUSTRALIA

## Abstract

Large brains in prey may select for adoption of anti-predator behavior that facilitates escape. Prey species with relatively large brains have been shown to be less likely to fall prey to predators. This results in the prediction that individuals that have been captured by predators on average should have smaller brains than sympatric conspecifics. We exploited the fact that Eurasian pygmy owls *Glaucidium passerinum* hoard small mammals and birds in cavities and nest-boxes for over-winter survival, allowing for comparison of the phenotype of prey with that of live conspecifics. In Northern Europe, main prey of pygmy owls are voles of the genera *Myodes* and *Microtus*, while forest birds and shrews are the most important alternative prey. Large fluctuations (amplitude 100-200-fold) in vole populations induce rapid numerical responses of pygmy owls to main prey populations, which in turn results in varying predation pressure on small birds. We found, weighed and measured 153 birds in food-stores of pygmy owls and mist-netted, weighed and measured 333 live birds of 12 species in central-western Finland during two autumns with low (2017) and high (2018) pygmy owl predation risk. In two autumns, individuals with large brains were captured later compared to individuals with small brains, consistent with the hypothesis that such individuals survived for longer. Avian prey of pygmy owls had smaller heads than live birds in autumn 2018 when predation risk by pygmy owls was high. This difference in head size was not significant in 2017 when predation risk by pygmy owls was reduced. Finally, avian survivors were in better body condition than avian prey individuals. These findings are consistent with the hypothesis that pygmy owls differentially prey on birds in poor condition with small brains. These findings are consistent with the hypothesis that predation risk imposed by pygmy owls on small birds in boreal forests varies depending on the abundance of the main prey (voles).

## Introduction

Predator-prey interactions often result in death of prey [1,2]. Therefore, there is strong selection for behaviors that facilitate escape from predators. Efficient predator evasion may in many cases require sophisticated behavior, although evasion behaviour can be improved through modifications to sensory-motor circuits. Predator evasion may in turn select for increased cognitive capacity and hence a larger brain. Eventually this may select for more efficient offense facilitating the capture and eventual killing of prey [3,4]. Hence, a range of different kinds of anti-predator behaviors that reduce or prevent successful predation have been described [3,4].

Assessment of the risk of predator attack and subsequent predator avoidance are achieved through cognitive mechanisms [5–7]. Several studies have hypothesized or shown that prey species with relatively small brains for their body size are disproportionately susceptible to predation [8–12]. For example, relatively large-headed female common eiders *Somateria mollissima* experienced higher survival than smaller-headed females during years with high predation risk, while the opposite was the case in years with lower mortality [13].

If individuals differ in cognitive abilities, this should have consequences for the ability to survive and hence for longevity. Indeed, Møller [14] showed for the barn swallow *Hirundo rustica* that individuals with larger head volumes were captured later during the spring because they took longer time to be captured. Furthermore, individuals that were more difficult to catch repeatedly were recaptured later during the season than small-headed individuals. For example, individuals of long-distance migratory barn swallows that arrived earlier to the breeding grounds after migration of more than 5000 km to southern Africa indeed had a larger head than individuals that arrived later [8]. Great tits *Parus major* with relatively larger heads for their body size had more exaggerated exploratory and novelty behavior [15]. Individuals with relatively larger heads for their body size were captured later during the day and later during the season. Hence head size may be related to personality.

**Fig 1:**
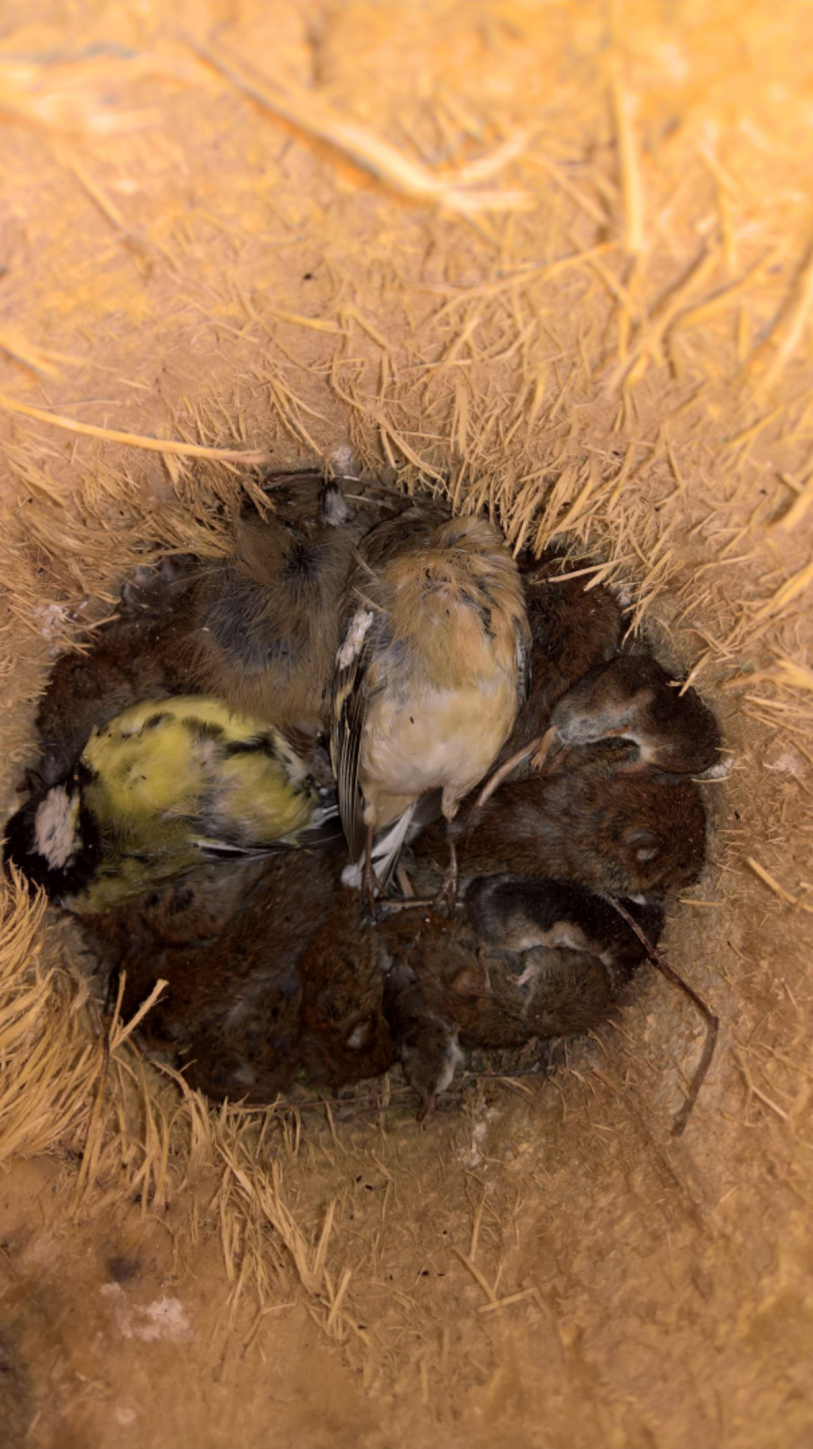
Food-store of pygmy owl in nest-box including male chaffinch, great tit, bank voles and common shrews *Sorex araneus*.

Predators that rely on food hoarding not only have to overcome the anti-predator defenses of prey and the associated cognitive bases [4,16,17], but also have to successfully recover any hoarded prey [18]. Many species of predators generally consume the brain of their prey before any other part of their body (e. g. [19–22], making it hard to estimate brain size of prey and non-prey. Exceptions to this general pattern in predator-prey interactions are some species of shrikes (*Lanius* spp., [23–25]) and the Eurasian pygmy owl *Glaucidium passerinum* [26], in which both males and females hoard large numbers of mammals and birds in nest-boxes and natural cavities from late autumn to early winter to survive the winter and facilitate successful reproduction [27–30] (Fig 1). This behavior allows for comparison of brain size of individuals that fell prey to this predator with that of conspecifics that were not captured. Pygmy owls are generalist predators, the main prey of which in boreal areas of Northern Europe are voles of the genera *Myodes* and *Microtus*. Most important alternative prey are shrews and small forest birds, particularly tit species (Paridae) both during the breeding season [31] and in winter [29,30]. Consumption of cached prey is widespread and virtually all such prey are consumed later. This allows to test whether the size of the brain differs between prey and non-prey.

In boreal regions of Northern Europe, populations of *Myodes* and *Microtus* voles fluctuate in high-amplitude three-year cycles (e.g. [32]). The amplitude (ratio of minimum to maximum density) is 100-200-fold [32]. Large fluctuations in vole populations induce rapid numerical responses of pygmy owls and other avian predators to main prey populations (e.g. [33,34]), which in turn results in densities of over-wintering pygmy owls are much larger in years of high vole abundances than in those of vole scarcity [29,30]. Therefore, the predation risk caused by pygmy owls on small birds in boreal forests varies greatly by being higher in years of abundant main prey than in years with a scarcity of such prey [30].

The objectives of this study were (1) to test if individuals that had been captured early in autumn or winter had smaller brains than individuals that survived autumn and winter. This was done by inclusion of body size and body mass as covariates with the control for the body size effect representing the body condition effect. (2) We predicted that prey species had smaller brains than non-prey of the same species, particularly in a year of abundant main prey when predation risk by pygmy owls is high. (3) We also tested whether this relationship was independent of body condition of prey and non-prey. We collected data at a long-term study site of pygmy owls in Finland for assessing these predictions in two autumns with varying predation risk imposed by pygmy owls [28–30,35].

## Materials and methods

### Study sites and methods

This study was conducted at 336 different sites in the Kauhava region, Central-Western Finland (63^o^N, 23^o^E) in a study area covering 1000 km^2^ including 240 forest patches, each with two nest-boxes for pygmy owls 80–100 m apart [28,29,35]. For a description of the nest boxes, see Terraube et al. [28], Morosinotto et al. [35] and Masoero et al. [29]. Pygmy owls use nest-boxes for food-hoarding and breeding, and no other avian predator is able to enter the boxes with small entrance hole (diameter 45 mm).

The study was conducted under permits from the Finnish authorities (ringing permit numbers 524 and 2588 from the Ringing Centre of Natural History Museum, Helsinki). Anesthesia, euthanasia, or any other kind of animal sacrifice was not part of the study.

We inspected all boxes carefully in late October to early December 2017 and 2018 with two visits per box, and found a total of 26 food-store sites with 316 prey items in 2017 and 93 food-store sites with 1822 prey items in 2018 (Table 1). Because most (>95%) prey items in food stores are hoarded whole (Fig 1), species identification of birds and mammals is feasible (Table 1).

**Table 1:** Number of main prey groups (voles of the genera *Myodes* and *Microtus*, shrews *Sorex araneus* and *S*. *minutus*, mice *Micromys minutus* and *Mus musculus*, and small birds) in food-stores of pygmy owls, number of food store sites and number of prey items per store-site, total number of owls trapped or encountered in the study area, as well as mean number of food-store sites per owl and median linear distance between food-store sites of individual owls (in parentheses) during autumns 2017 and 2018.

Year	Voles	Shrews	Mice	Birds	Total	No. of store sites	No. of prey per store site	No. of owls	Mean no. (distance) of store-sites
2017	165	83	24	44	316	26	12.2	16	1.3 (2300 m)
2018	1520	52	104	146	1822	93	19.6	35	2.8 (1750 m)

To obtain density estimates of over-wintering pygmy owls (Table 1), and to estimate the level of predation risk imposed by pygmy owls on small birds, pygmy owls were captured at the food-store sites with nest-box traps (a model of the box equipped with a swing door) [28,29]. Captured individuals were marked with an aluminum leg-ring and provided with an RFID tag, a microchip with a unique code [36]. A majority of owls (81%) at food-stores were captured with box-traps [29], but additional data on encounters of individual owls at food-stores were collected by setting up the antenna of the RFID-reader around the entrance hole of the store-box. The antenna and the reader were positioned when the food store was found, when capture of the owl with the nest-box trap was unsuccessful.

We weighed avian prey with a Pesola spring balance to the nearest 0.5 g and measured flattened wing length of avian prey to the nearest mm. We removed the heads of avian prey and measured maximum head length, beak length to feathering, maximum head width and maximum head height to the nearest 0.01mm. The corpses were subsequently returned to the original food-store boxes in order not to remove crucial resources from the over-wintering pygmy owls. We measured head length, width and height three times to estimate repeatability as the intra-class correlation coefficient [37]. Head volume was highly repeatable at 0.945, *F* = 688.53, df = 1, 771, *r*^2^ = 0.89, *P* < 0.0001). Specimens were sexed and aged according to Svensson [38].

We captured live birds with mist-nets at seven bird feeding sites around Kauhava in winter 2017–2018 and 2018–2019 for comparison of their morphology with that of individuals that had been killed by the pygmy owls. Of the seven mist-net sites, two were in the eastern part, two in the middle, two in the western and one in the northern part of the pygmy owl nest-box area (1000 km^2^). These individuals were weighed and measured as described above to estimate head volume.

We can infer from our repeated mist-net captures of birds that we managed to capture more than 90% of all individuals during our repeated capture sessions. We can also conclude that this high capture rate was due to our study birds being resident during our study in midwinter. This finding is similar to that of another study of resident great tits in France showing only negligible movements during winter and early spring [15].

Head volume was estimated as 4/3 x π x (head length–beak length) / (2 x head width / 2 x head height / 2) [14]. Head volume times 1.034 g/cm^3^ provides an estimate of brain mass (J. Erritzøe unpublished data).

Head volume and brain size are strongly positively correlated in great tits (*F* = 242.24, d.f. = 1,19, adjusted *r*^2^ = 0.93, *P* < 0.0001, estimate (SE) = 0.289 (0.019)), [15]), barn swallows (*F* = 1459.41, d.f. = 1,8, adjusted *r*^2^ = 0.99, *P* < 0.0001, estimate (SE) = 0.190 (0.005) [14], and common eider (*F* = 39.21, d.f. = 1,13, adjusted *r*^2^ = 0.73, *P* < 0.0001, estimate (SE) = 0.199 (0.030) [13]). Hence, we could transform head size to brain size using the equation log Brain size = 0.7494 (SE = 0.0132) log Head volume– 2.6374 (SE = 0.0483), *F* = 3217.24, d.f. = 1, 132, *r*^2^ = 0.96, *P* < 0.0001.

Abundances of voles have been estimated by snap-trappings each year in mid-May and in late September in the western and middle part of the study area during 1973–2019. Both in the western and middle part, four plots were sampled in May and September (i.e. cultivated field, abandoned field, spruce forest, pine forest; see Korpimäki et al. [32] for more details on trapping methods and vole cycles). Fifty to 60 Finnish metal mouse snap traps were set at 10 m intervals in vole runways in each plot, and they were checked once a day for three days. Thus, the area of a sample plot ranged from 0.5 to 0.6 ha. The results from 3-night trapping periods were pooled and standardized to the pooled number of bank and *Microtus* voles caught per 100 trap nights (which was later termed density estimate of voles).

### Statistical analyses

We emphasize two aspects of statistical components of the study. First, the study was based on prey that varied slightly in size. The pygmy owls and we captured prey in the size range of 9 to 32 g. Date, body mass and morphological characters were log_10_-transformed in order to obtain normally distributed variables to fulfill the criteria for the statistical analyses. Therefore, there was a high degree of overlap in body size, body mass and head volume. This implies that there was no need for allomeric analyses. Second, we did not conduct phylogenetic comparative analyses based on statistical methods as described [39], because a unit of food from one prey species does not deviate from a unit of food from another. Thus 10 grams of food from a *Microtus* vole is equivalent with 10 grams of food from say a great tit or a bullfinch. Given the small number of species compared, it is unlikely that any of these methods could be applied successfully.

We calculated repeatability as the intra-class correlation coefficient [37].

We used a GLMM (Generalized Linear Mixed Model) with date as the response variable (the date when an individual was last captured with larger values reflecting greater difficulty of catching a specific individual resulting in a later date) (Table 2). The GLMM for capture date had a normally distributed response variable with an identity link function and storage year (fixed factor), prey or not (fixed factor), head volume (covariate), and prey by storage year interaction, species as a random effect and food-store site or mist-net site as random effects. We used the REML variance component estimates with 95% confidence intervals.

**Table 2:** Generalized Linear Mixed model (GLMM) of the relationship between date when the individual hoarded avian prey was last found and storage year (fixed effect), whether an individual was a prey or not (fixed effect), head volume (covariate), species (random effect), food-store or mist-net site (random effect) and the interaction between whether individual birds were prey of pygmy owls (fixed effect) and storage year (fixed effect). Sample size was 486.

Term	*F*	d.f.	*P*	Estimate	SE
Intercept	0.490	114.5	0.483	-2486.663	3530.645
Storage year	0.513	114.5	0.475	1.253	1.749
Prey	337.866	28.7	0.046	27.787	1.512
Head volume	4.223	46.1	0.046	16.974	8.260
Prey [No] x Storage year	111.7		< 0.0001	11.508	1.715
Random Variance effect ratio	Variance component		SE	95% Lower	95% Upper
Species 0.167	18.284		14.360	-9.861	46.429
Site 0.293	32.195		12.788	7.130	57.259

We used a GLMM with log_10_-transformed head volume as the normally distributed response variable with an identity link function, wing length (covariate), species (random factor) to account for differences in sample size among species, and whether individual birds were prey of pygmy owls or alive (fixed effect). We tested if head volume of avian prey was smaller than head volume of live captures using a Wilcoxon matched-pairs signed-ranks test based on mean values for the different species and sample size as a weighting factor. We used Wilcoxon matched-pairs signed-ranks tests rather than GLMM for some of the analyses because the data did not fulfill the criteria for GLMM. Finally, we tested if head volume of birds was related to whether individuals were prey or not prey independent of their body condition by inclusion of body mass and wing length as measures of structural body size. Wing length was not a significant predictor in any of these analyses, and it was subsequently excluded from all analyses. We used the REML variance component estimates with 95% confidence intervals. All statistical analyses were made with JMP [40].

## Results

The density estimate of voles available in the field was relatively low in autumn 2017 (pooled number of bank and *Microtus* voles 5.3 per 100 trap-nights), but three times higher in autumn 2018 (15.9 voles per 100 trap nights). The marked increase in vole abundance induced rapid numerical response of pygmy owls: the number of individual pygmy owls encountered at food-stores of the study area in autumn 2018 was twice as high as that of conspecifics in autumn 2017 (Table 1). In addition, 46% (out of 35) of pygmy owls had more than one food-store site in 2018, whereas the corresponding proportion was only 31% (out of 16) in 2017. The mean number of food-store sites per owl was also twice as large in 2018 (range 1–6) as in 2017 (1–2; Table 1). Multiplying the number of owls present in the study area with the number of food-store sites, where owls were encountered, resulted in approximately five times more food-store sites being visited by pygmy owls in 2018 than in 2017 (98 vs. 21, in 2018 five food-store sites were visited by two owls, while in 2017 none were visited twice; see Table 1). These food-store sites were on average 2207 m apart during 2017–2018. Therefore, we can conclude that the predation risk imposed by pygmy owls on small birds in the study area was considerably higher in 2018 than in 2017. This is further supported by pygmy owls killing and storing three times as many birds in 2018 as in 2017 (Table 1).

**Fig 2:**
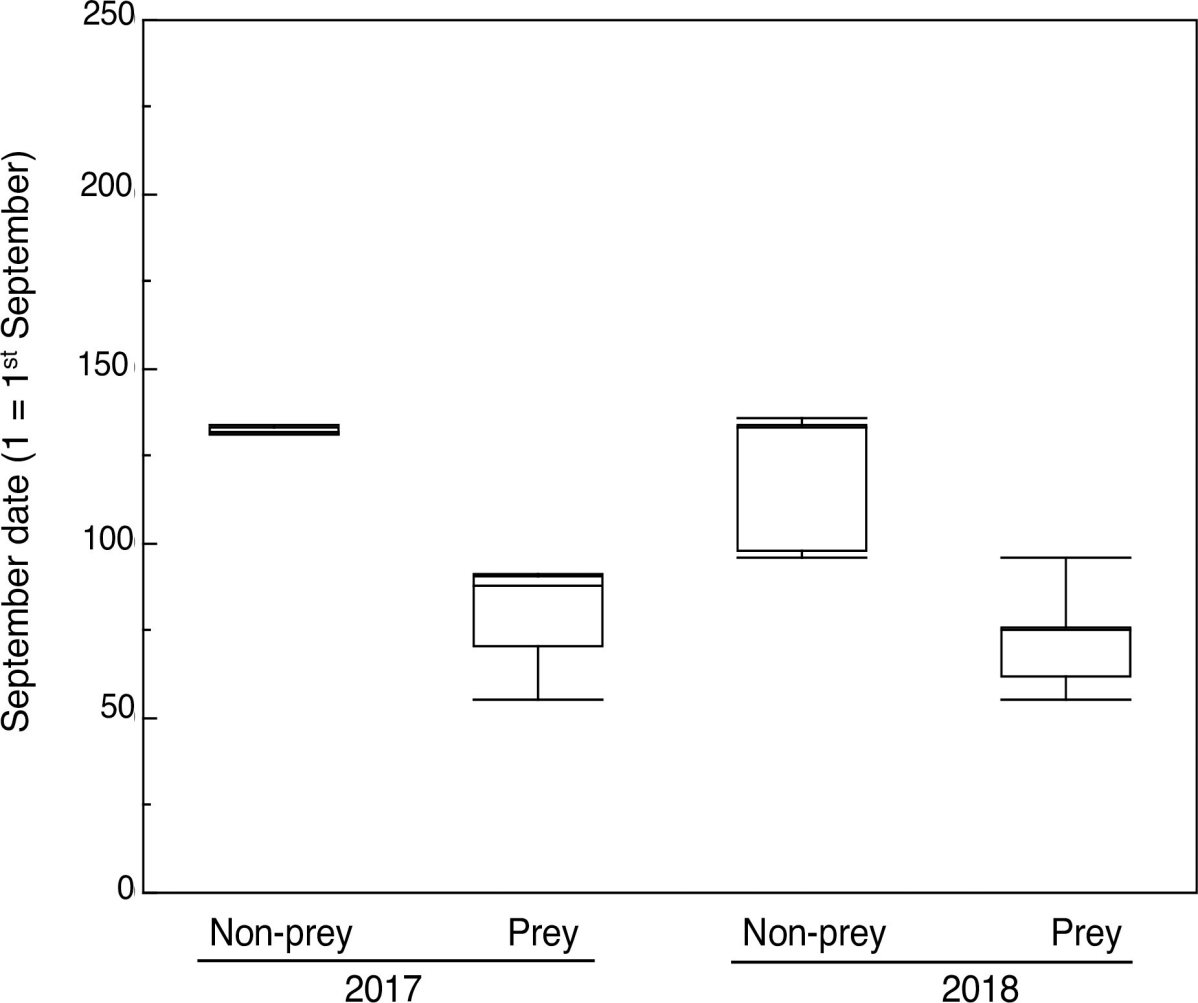
Box plot of September date when prey and non-prey birds were recorded during autumn 2017 and 2018. Box plots show median, quartiles and 5- and 95-percentiles.

We found corpses of 13 bird species in the 152 food store sites of pygmy owls, and we were able to capture individuals of 12 of these species at seven mist net sites distributed over the large study area during autumn 2017–2018 (Fig 2): long-tailed tit *Aegithalos caudatus* (1 hoarded specimen, none mist-netted), common redpoll *Acanthis flammea* (22 hoarded specimens, 9 mist-netted), great tit (19, 141), blue tit *Cyanistes caeruleus* (29, 66), coal tit *Periparus ater* (3, 11), crested tit *Poecile cristatus* (20, 19), willow tit *Poecile montanus* (35, 41), Eurasian tree-creeper *Certhia familiaris* (2, 7), goldcrest *Regulus regulus* (16, 2), yellowhammer *Emberiza citrinella* (3, 6), greenfinch *Carduelis chloris* (1, 2), chaffinch *Fringilla coelebs* (1, 1) and bullfinch *Pyrrhula pyrrhula* (1, 4). The conclusion was the same when species with only one specimen was excluded (paired t-test, *t* = 4.33, df = 8, *P* = 0.0025, Wilcoxon matched pairs-signed ranks test, W = -48907.0, *P* < 0.0001). Finally, we reached a similar conclusion when species with 2–5 specimens were excluded.

Avian prey were captured by pygmy owls later in 2017 than in 2018, and prey with larger heads were found later in the year than live birds (Fig 2, Table 2).

**Fig 3:**
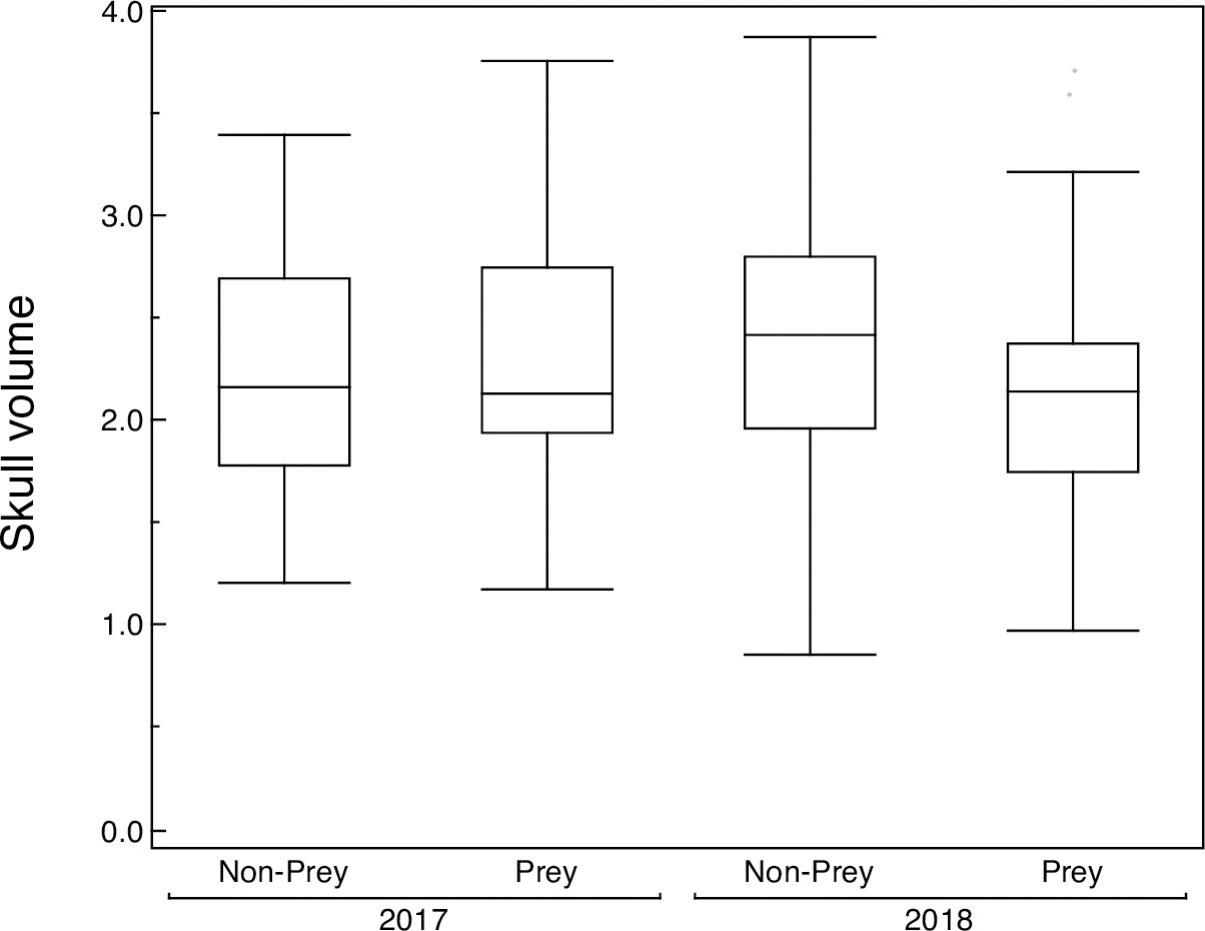
Box plot of the head volume of prey and non-prey recorded during autumn 2017 and 2018. Box plots show median, quartiles and 5- and 95-percentiles.

Finally, there was a significant interaction between storage year and whether individuals were prey (Fig 3, Table 3). Hence head volume of live birds was larger than that of prey, but only in 2018 (Fig 3, Table 3).

**Table 3:** Generalized Linear Mixed model (GLMM) of the relationship between log^10^-transformed head volume of birds, and storage year (fixed effect), whether an individual was prey or not (fixed effect), body mass (covariate), wing length (covariate), storage year x prey interaction, species (random effect) and food-store or mist-net site (random effect). Sample size was 486.

Term	*F*	d.f.	*P*	Estimate	SE
Intercept	825	24.44	< 0.0001	2.77	0.10
Storage year [2017]	0.01	13.87	0.93	0.000	0.003
Prey [Non-prey]	11.21	0.73	0.25	-0.010	0.003
Sex	4.90	262.2	0.028	-0.007	0.028
Body mass	96.03	184.4	< 0.0001	0.712	0.073
Storage year x Prey	13.03	16.9	0.002	0.012	0.003
Wing length	12.95	228.7	0.0004	0.712	0.073
Random Variance	Variance component		SE	95% Lower	95% Upper
Effect ratio					
Species 4.02	0.003		0.002	0.0000001	0.000001
Site 0.160	0.0000000002		0.00000003	0.0000002	0.000001

**Fig 4:**
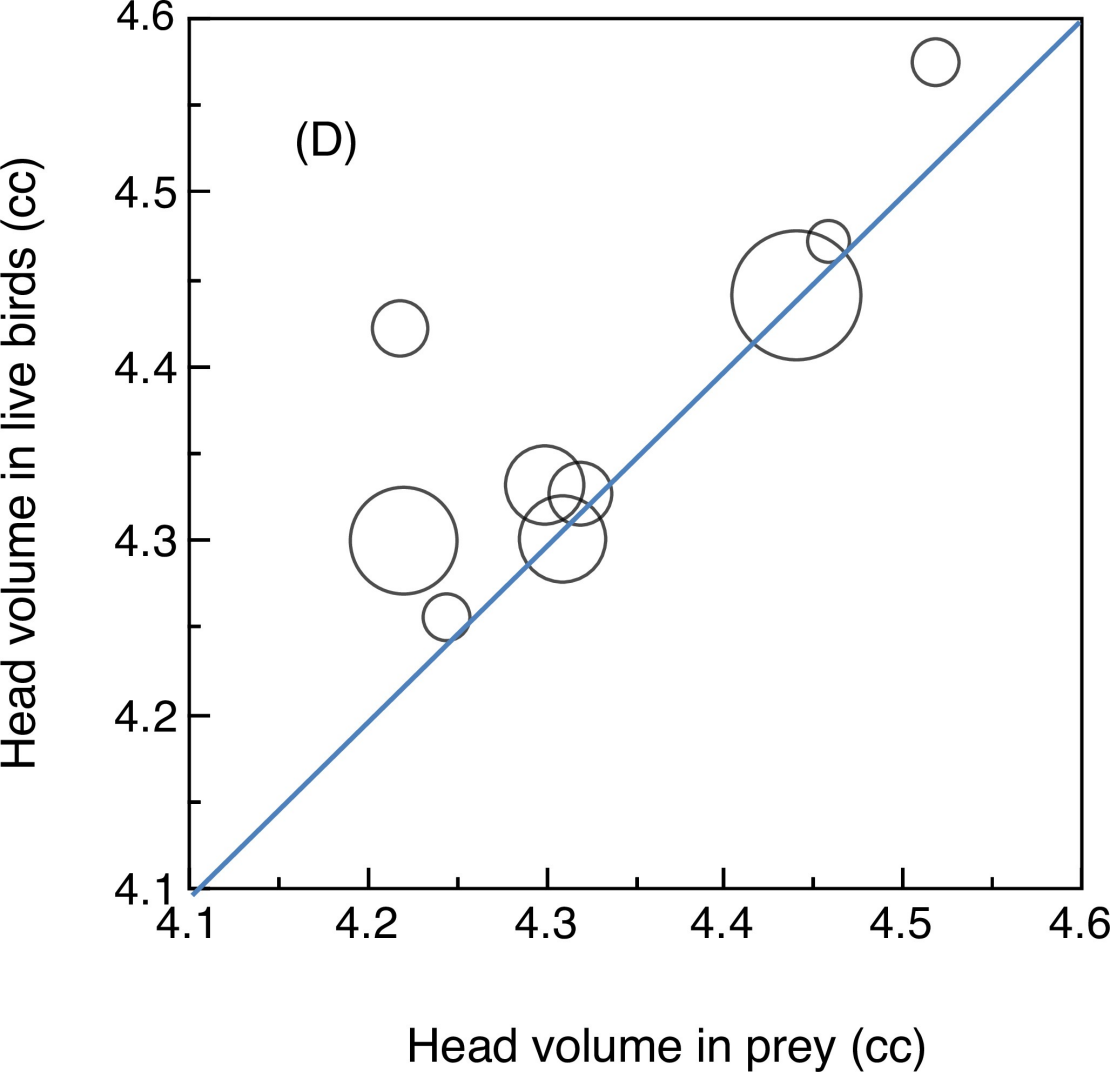
Head volume of live avian prey species in relation to head volume of prey. The line is Y = X, showing that live birds consistently had larger head volumes than prey. The size of circles is proportional to sample size, and one circle represents one species. This analysis included 12 species since there were no chaffinches in the samples of live prey birds.

Relative skull size of 151 individuals of the 12 bird species killed by pygmy owls was on average 5% smaller than that of live conspecifics of the same species (Fig 4, Wilcoxon matched-pairs signed-ranks test, *W* (Wilcoxon test statistic) = 32,000, *P* = 0.0093), even when estimates were weighted by sample size (Fig 4; *W* (Wilcoxon test statistic = 56,372, *P* < 0.001). In fact, prey of 9 out of 12 prey species for pygmy owls had smaller brains than non-prey (Fig 4).

## Discussion

We conducted by far the largest study of the link between head volume (a reliable measure of brain size [13–15]; J. Erritzøe and A. P. Møller), and the risk of predation for 486 small birds during two winters in 336 study sites dispersed across a 1000 km^2^ study area in Finland. The difference in skull size between prey and non-prey of 2–7% is similar to average effect sizes in studies of ecology and evolution [41]. We showed a correlation between predation avoidance and skull size in birds, but only when the predation risk was high. Birds with larger brains may simply be better foragers and therefore be in better body condition than birds with smaller brains. This hypothesis requires further study.

How can prey with small brains be maintained in populations if predators disproportionately often capture and consume individuals with small brains? Adverse environmental conditions may impair normal brain development. For example, a protein-deficient diet may reduce brain development and impair learning ability as shown experimentally for zebra finches *Taeniopygia guttata* [42]. Here we showed that individual birds with small brains for their body size are more common in poor habitats or in years with particularly adverse environmental conditions. These hypothetical relationships showed that head size was correlated with body mass and body size. These associations may arise due to the effects of the cognitive buffer hypothesis [43].

Prey with large head volume took longer time to capture. Hence birds were more difficult to capture if head volume was large. That was the case when predation risk by pygmy owls was high, independent of statistical control for body mass, body size, sex and age. This result is consistent with findings for the barn swallow showing that individuals with larger heads are captured later, but also recaptured less often [14]. Similar findings have been reported for fish [44–46].

This study revealed that under risky conditions birds with small heads were more likely to be killed by predators than individuals with large heads. This effect was partially mediated by body condition as reflected by body size and body mass. Predation risk imposed by pygmy owls on alternative prey (small birds) in boreal forests varies depending on the abundance of main prey (voles). We suggest that large brains and potentially cognitive skills of small birds may improve anti-predator behavior and thus facilitate survival under harsh climatic conditions. This suggestion is supported by earlier results that flight initiation distances of birds are positively correlated with brain size and the size of the cerebellum at both intraspecific and interspecific levels [6]. A majority of the small birds killed by pygmy owls were comprised of tits (Paridae). In winter, tits, goldcrests and tree-creepers aggregate in mixed-species flocks in boreal forests and deliver intense interspecific alarm calls against predators including pygmy owls (e.g. [47]). Many forest birds also often show interspecific collaborative mobbing behavior against pygmy owls [48], and such behavior may facilitate escape from predators.

## Supporting information

S1 Table (PDF)

S2 Table (PDF)

S3 Table (PDF)

S1 Data (XLSX)
