# Supplementary material for: Predation risk in relation to brain size in alternative prey of pygmy owls varies depending on the abundance of main prey
Source: PLoS One. 2020 Sep 11;15(9):e0236155. doi: 10.1371/journal.pone.0236155 (PMC7485837; doi:10.1371/journal.pone.0236155)
Supplement: S1 Table — (PDF) [file pone.0236155.s001.pdf]

Response log Day 1st Sep=1

Summary of Fit

RSquare	0.83027
RSquare Adj	0.828445
Root Mean Square Error	0.051685
Mean of Response	2.016713
Observations (or Sum Wgts)	471

Parameter Estimates

Term	Estimate	Std Error	DFDen	t Ratio	Prob>ltl
Intercept	1.6730554	0.168518	317.2	9.93	<.0001*
Store yr[2017]	0.0233599	0.002881	452.8	8.11	<.0001*
Wing Length	-0.002667	0.001129	432.7	-2.36	0.0186*
Weight	0.0100873	0.002198	131.7	4.59	<.0001*
Capture method[mist-net]	0.1075281	0.003777	315.3	28.47	<.0001*
log Volume	0.1144861	0.048737	393.6	2.35	0.0193*

REML Variance Component Estimates

Random Effect	Var Ratio	Var Component	Std Error	95% Lower	95% Upper	Pct of Total
Species	1.2612704	0.0033693	0.0027485	-0.002018	0.0087563	55.777
Locality SITE	8.3824e-7	2.2392e-9	3.4552e-9	-4.533e-9	9.0112e-9	0.000
Residual		0.0026714	0.0001788	0.0023529	0.0030596	44.223
Total		0.0060407	0.0027338	0.0029285	0.0189283	100.000

-2 LogLikelihood = -1371.141997

Note: Total is the sum of the positive variance components.

Total including negative estimates = 0.0060407

Fixed Effect Tests

Source	Nparm	DF	DFDen	F Ratio	Prob > F
Store yr	1	1	452.8	65.7376	<.0001*
Wing Length	1	1	432.7	5.5812	0.0186*
Weight	1	1	131.7	21.0594	<.0001*
Capture method	1	1	315.3	810.6144	<.0001*
log Volume	1	1	393.6	5.5181	0.0193*

Effect Details

Store yr

Least Squares Means Table

Level	Least Sq Mean	Std Error
2017	2.0234889	0.02024584
2018	1.9767691	0.02004204

Response log Day 1st Sep=1

Effect Details

Wing Length

Weight

Capture method

Least Squares Means Table

Level	Least Sq Mean	Std Error
mist-net	2.1076571	0.02019124
Pygmy owl	1.8926009	0.02038672

log Volume

Species

Least Squares Means Table

Level	Least Sq Mean	Std Error
carmea	2.0134336	0.01152571
cerfam	2.0119804	0.01982846
embcit	1.8951711	0.03011722
parate	2.0335504	0.01591883
parcae	2.0068392	0.00711190
parcri	1.9879574	0.01053267
parmaj	1.9524894	0.00974594
parmon	2.0008302	0.00880304
regreg	2.0989094	0.02170247

Locality SITE

Prediction Profiler

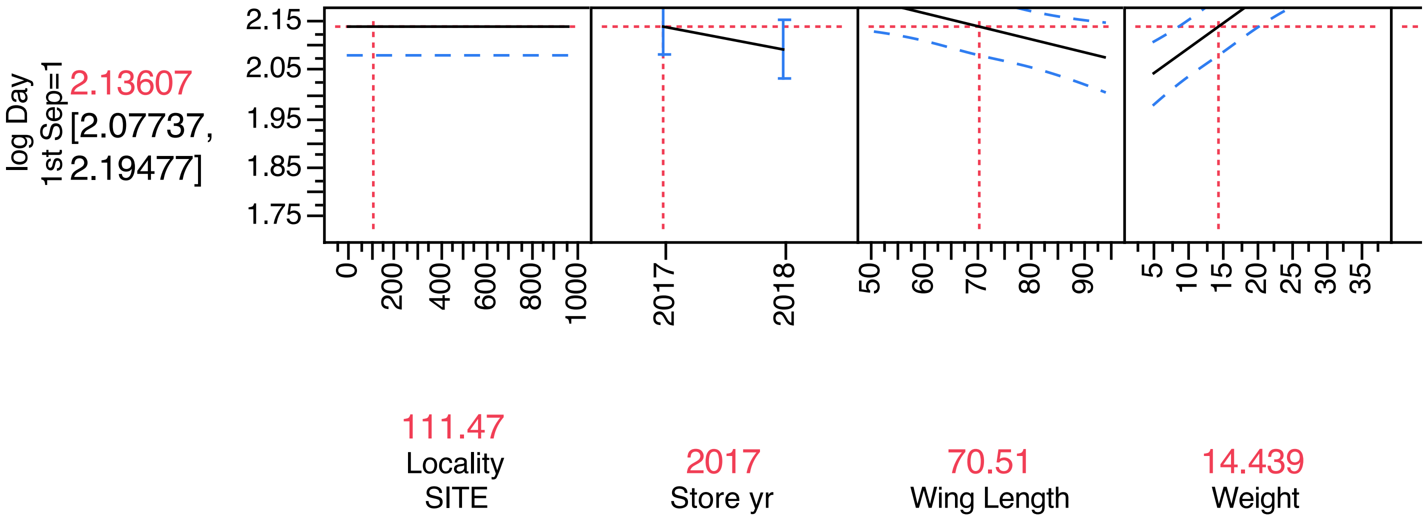

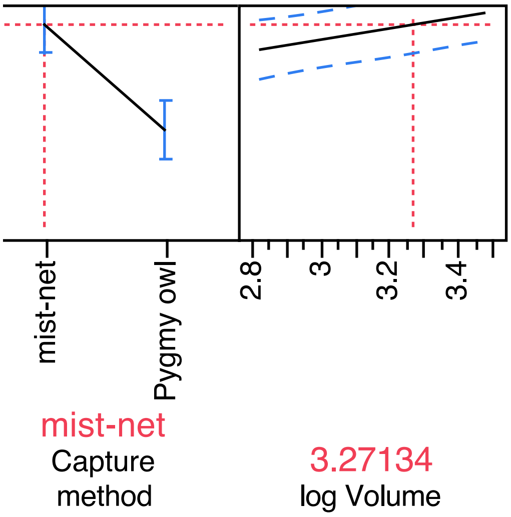
mist-net
Pygmy owl
2.8
3
3.2
3.4
mist-net
Capture method
3.27134
log Volume
