# Supplementary material for: Predation risk in relation to brain size in alternative prey of pygmy owls varies depending on the abundance of main prey
Source: PLoS One. 2020 Sep 11;15(9):e0236155. doi: 10.1371/journal.pone.0236155 (PMC7485837; doi:10.1371/journal.pone.0236155)

Response log Volume

Summary of Fit

RSquare	0.809937
RSquare Adj	0.808705
Root Mean Square Error	0.049729
Mean of Response	3.27203
Observations (or Sum Wgts)	467

Parameter Estimates

Term	Estimate	Std Error	DFDen	t Ratio	Prob>ltl
Intercept	3.0219681	0.042312	15.91	71.42	<.0001*
Capture method[mist-net]	-0.014896	0.003327	16.21	-4.48	0.0004*
Age[ad]	-0.0043	0.002366	455.4	-1.82	0.0698
Weight	0.0138091	0.001836	267	7.52	<.0001*

REML Variance Component Estimates

Random Effect	Var Ratio	Var Component	Std Error	95% Lower	95% Upper
Species	4.1724065	0.0103182	0.0052463	3.5578e-5	0.0206007
Locality SITE	9.4525e-8	2.338e-10	5.961e-10	-9.34e-10	1.402e-9
Residual		0.0024729	0.0001641	0.0021804	0.0028289
Total		0.0127911	0.0052488	0.0065587	0.0350805

-2 LogLikelihood = -1401.069386

Note: Total is the sum of the positive variance components.

Total including negative estimates = 0.0127911

Fixed Effect Tests

Source	Nparm	DF	DFDen	F Ratio	Prob > F
Capture method	1	1	16.21	20.0468	0.0004*
Age	1	1	455.4	3.3038	0.0698
Weight	1	1	267	56.5428	<.0001*

Effect Details

Capture method

Least Squares Means Table

Level	Least Sq Mean	Std Error
mist-net	3.2080818	0.03418177
Pygmy owl	3.2378734	0.03436037

Age

Least Squares Means Table

Pct of Total
80.667
0.000
19.333
100.000

Response log Volume

Effect Details

Age

Least Squares Means Table

Level	Least Sq Mean	Std Error
ad	3.2186772	0.03421434
juv	3.2272781	0.03416858

Weight

Species

Least Squares Means Table

Level	Least Sq Mean	Std Error
carmea	3.1650079	0.00928581
cerfam	3.1435145	0.02014170
embcit	3.1036857	0.03222639
parate	3.2829670	0.01561568
parcae	3.2368164	0.00684031
parcri	3.3497131	0.00948322
parmaj	3.3169664	0.00937451
parmon	3.3201361	0.00813398
regreg	3.0879915	0.01951041

Locality SITE

Prediction Profiler

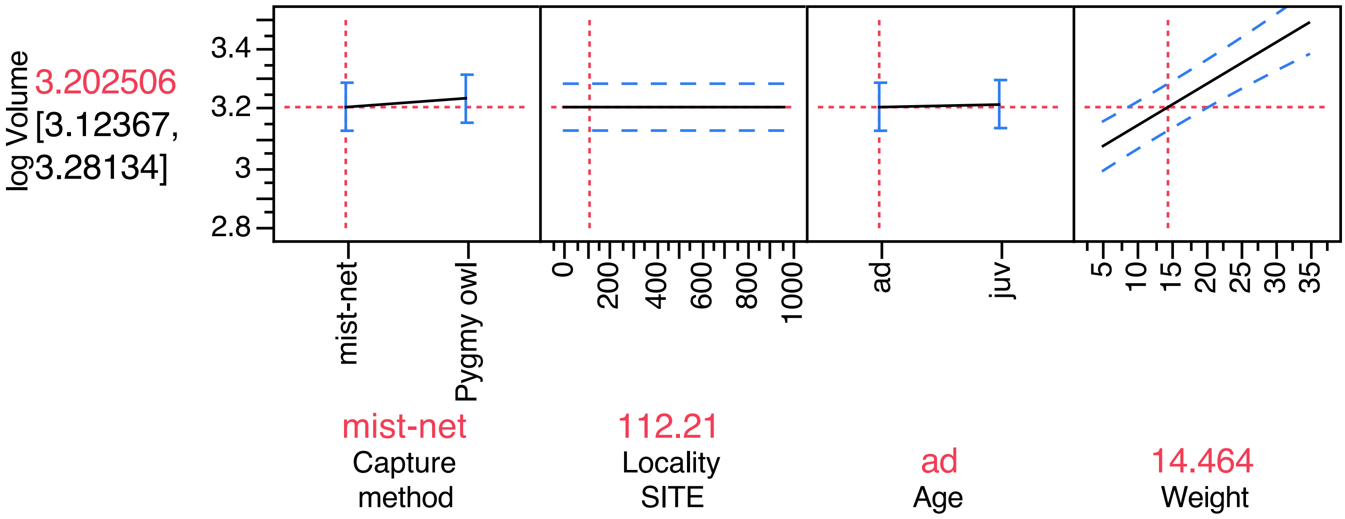

Supplement: S2 Table — (PDF) [file pone.0236155.s002.pdf]
