# Supplementary material for: Predation risk in relation to brain size in alternative prey of pygmy owls varies depending on the abundance of main prey
Source: PLoS One. 2020 Sep 11;15(9):e0236155. doi: 10.1371/journal.pone.0236155 (PMC7485837; doi:10.1371/journal.pone.0236155)
Supplement: S3 Table — (PDF) [file pone.0236155.s003.pdf]

Response Volume

Summary of Fit

RSquare	0.12857
RSquare Adj	0.10294
Root Mean Square Error	0.322128
Mean of Response	1.474484
Observations (or Sum Wgts)	176

Parameter Estimates

Term	Estimate	Std Error	DFDen	t Ratio	Prob>ltl
Intercept	0.7127783	0.287772	169.4	2.48	0.0142*
Store yr[2017]	0.1416328	0.050141	170	2.82	0.0053*
Capture method[Pygmy owl]	0.0730016	0.093733	169.6	0.78	0.4372
Body length	0.0098886	0.003237	169.2	3.05	0.0026*
Sex[f]	0.0030208	0.025133	169.2	0.12	0.9045
Age[ad]	-0.087097	0.075566	169.8	-1.15	0.2507

REML Variance Component Estimates

Random Effect	Var Ratio	Var Component	Std Error	95% Lower	95% Upper
Species	0	0	0	0	0
Locality SITE	3.488e-7	3.6193e-8	6.1951e-8	-8.523e-8	1.5761e-7
Residual		0.1037661	0.0112883	0.0847628	0.1299995
Total		0.1037662	0.0112883	0.0847628	0.1299995

-2 LogLikelihood = 128.05398878

Note: Total is the sum of the positive variance components.
Total including negative estimates = 0.1037662
Warning: Random effects are confounded with the fixed effects.
Non-estimable variance components have been zeroed.
Estimates and test statistics are biased and of questionable value.

Fixed Effect Tests

Source	Nparm	DF	DFDen	F Ratio	Prob > F
Store yr	1	1	170	7.9790	0.0053*
Capture method	1	1	169.6	0.6066	0.4372
Body length	1	1	169.2	9.3297	0.0026*
Sex	1	1	169.2	0.0144	0.9045
Age	1	1	169.8	1.3285	0.2507

Pct of Total
0.000
0.000
100.000
100.000
